# Jet-Cooled Phosphorescence Excitation Spectrum of the T_1_(n,π*) ← S_0_ Transition of 4*H*-Pyran-4-one

**DOI:** 10.1021/acs.jpca.3c01059

**Published:** 2023-04-17

**Authors:** Sean W. Parsons, Devon G. Hucek, Piyush Mishra, David F. Plusquellic, Timothy S. Zwier, Stephen Drucker

**Affiliations:** † Department of Chemistry and Biochemistry, University of Wisconsin-Eau Claire, 105 Garfield Avenue, Eau Claire, Wisconsin 54701, United States; § Department of Chemistry, Purdue University, 560 Oval Drive, West Lafayette, Indiana 47907, United States; ⊥ Applied Physics Division, National Institute of Standards and Technology, 325 Broadway Avenue, Boulder, Colorado 80305, United States

## Abstract

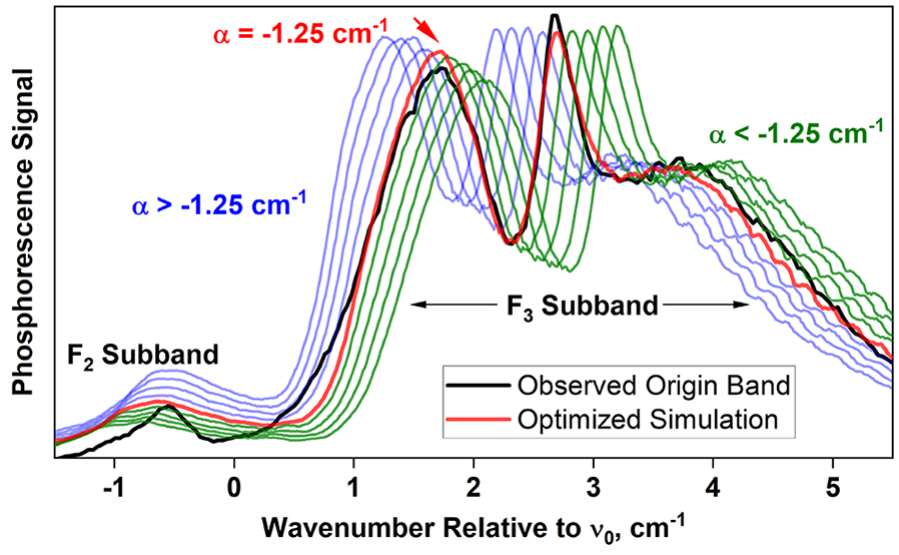

The 4*H*-pyran-4-one (4PN) molecule is a cyclic conjugated enone with spectroscopically accessible singlet and triplet (n,π*)excited states. Vibronic spectra of 4PN provide a stringent test of electronic-structure calculations, through comparison of predicted vs measured vibrational frequencies in the excited state. We report here the T_1_(n,π*) ← S_0_ phosphorescence excitation spectrum of 4PN, recorded under the cooling conditions of a supersonic free-jet expansion. The jet cooling has eliminated congestion appearing in previous room-temperature measurements of the T_1_ ← S_0_ band system and has enabled us to determine precise fundamental frequencies for seven vibrational modes of the molecule in its T_1_(n,π*) state. We have also analyzed the rotational contour of the 0_0_^0^ band, obtaining experimental values for spin–spin and spin-rotation constants of the T_1_(n,π*) state. We used the experimental results to test predictions from two commonly used computational methods, equation-of-motion excitation energies with dynamical correlation incorporated at the level of coupled cluster singles doubles (EOM-EE-CCSD) and time-dependent density functional theory (TDDFT). We find that each method predicts harmonic frequencies within a few percent of observed fundamentals, for in-plane vibrational modes. However, for out-of-plane modes, each method has specific liabilities that result in frequency errors on the order of 20–30%. The calculations have helped to identify a perturbation from the T_2_(π,π*) state that leads to unexpected features observed in the T_1_(n,π*) ← S_0_ origin band rotational contour.

## Introduction

**Figure 1:**
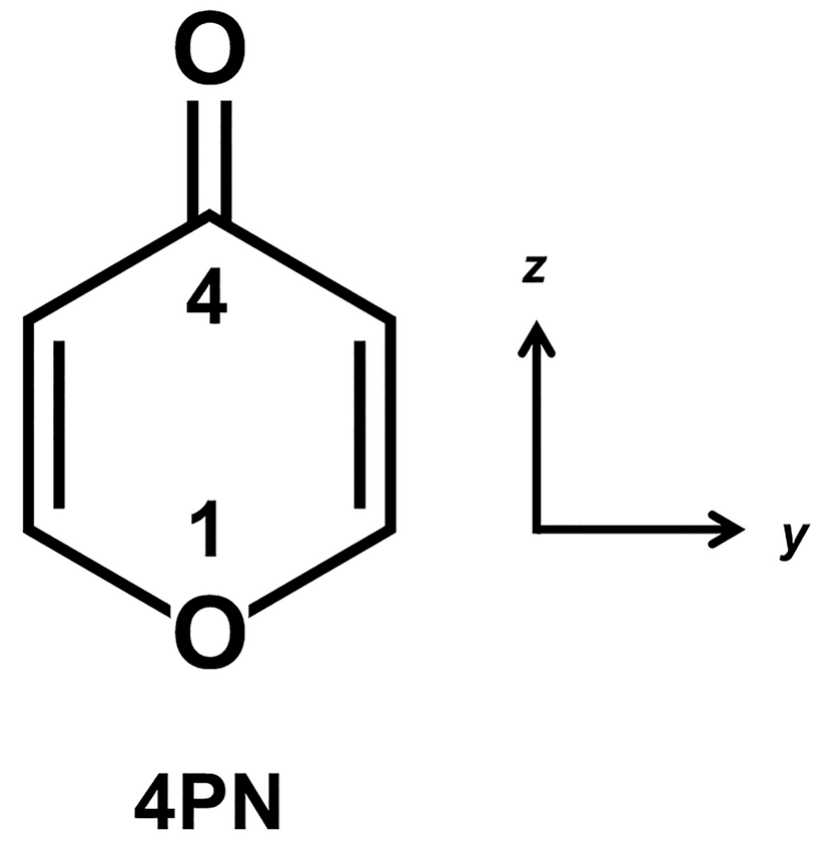
Structural formula of 4*H*-pyran-4-one (4PN), along with the molecule-fixed coordinate system used here. The molecule has a *C*_2*v*_ planar equilibrium geometry in its ground and T_1_(n,π*) states.

In this paper, we report the vibrationally resolved T_1_(n,π*) ← S_0_ phosphorescence excitation spectrum of 4*H*-pyran-4-one (4PN, Figure 1), recorded under the cooling conditions of a supersonic free-jet expansion. The 4PN molecule is a prototypical cyclic enone and serves as a model for investigating the π* ← n chromophore in α,β-unsaturated carbonyls. Triplet excited states of unsaturated molecules can play key roles in photochemistry,^1^ combustion,^2^ and energy storage.^3^ Triplet excited states are generally long-lived, enhancing their abiliity to participate in these processes. The triplet-state lifetimes are long because radiative decay to the singlet ground state is spin-forbidden. However, the attribute of spin-forbiddenness makes it difficult to study triplet excited states spectroscopically when starting with a molecule in its singlet ground state.

In previous work,^4,5^ we have overcome this limitation by using the highly sensitive cavity ringdown^6^ (CRD) absorption technique at room temperature or phosphorescence excitation (PE) spectroscopy under jet-cooled conditions^7^ to measure singlet–triplet spectra. The jet-cooled PE technique was pioneered in the 1980s to study triplet excited states of small-^8^ and medium-sized^9^ molecules.

Recently, we measured the T_1_ ← S_0_ vibronic band system of 4PN vapor using CRD spectroscopy.^4^ Under the ambient conditions of that experiment, the 4PN spectrum is congested with vibronic hot bands (*v*″ > 0) associated with both the T_1_(n,π*) ← S_0_ and the higher-energy S_1_(n,π*) ← S_0_ transition. The present jet-cooled PE approach has suppressed nearly all of the vibronic hot bands and significantly narrowed the rotational contours of the observed T_1_ ← S_0_ cold bands (*v*″ = 0). These simplifications have allowed us to confirm (or correct, in certain cases) vibronic assignments we made previously.^4^ We observe effective rotational temperatures as on the order of 5 K in the jet expansion, and this has permitted us to analyze the rotational contour of the T_1_ ← S_0_ origin (0_0_^0^) band. The analysis provides estimates for spin–spin and spin–rotation constants^10^ of the T_1_(n,π*) state of 4PN.

We have used the vibronic assignments in the jet-cooled PE spectrum to ascertain fundamental frequencies for most of ring vibrational modes in the T_1_(n,π*) state of 4PN. The measured frequencies have allowed us to evaluate computational methods for treating excited states. Two accessible and generally reliable techniques are equation-of-motion excitation energies with dynamical correlation incorporated at the level of coupled cluster singles doubles (EOM-EE-CCSD)^11,12^ and time-dependent density functional theory (TDDFT).^13^ At a moderate computational cost, the EOM-EE-CCSD *ab initio* method can perform nearly as well^14−16^ as “gold-standard” methods such as complete active-space second-order perturbation theory (CASPT2)^17^ or closed shell coupled cluster singles and doubles with perturbative triples (CC3).^18^ The gold standards are too expensive for treating molecules having more than a few heavy atoms (such as 4PN), but EOM-EE-CCSD is feasible for excited-state geometry optimization and harmonic frequency calculations of monocylic molecules.^4,19^

For even larger molecules, the very inexpensive TDDFT method is in widespread use. For example, accurate TDDFT calculations of vibronically resolved spectra are available for molecules such as conjugated dyes containing several fused rings.^20^

Spectroscopic examination of 4PN—particularly its T_1_(n,π*) excited state—offers a rigorous test of the EOM-EE-CCSD and TDDFT methods. The 4PN molecule has several different functional groups, along with conjugation, and these characteristics can affect the molecular orbitals in subtle ways. Moreover, configuration interaction between T_1_(n,π*) and other states in the triplet manifold is important, as evidenced by significant differences in T_1_(n,π*) vs S_1_(n,π*) fundamental frequencies^4,21^ measured for certain corresponding modes. TDDFT does not treat static correlation explicitly and is not expected to reproduce these frequency differences as well as EOM-EE-CCSD can. Though 4PN is near the upper size limit for EOM-EE-CCSD frequency calculations (with triple-ζ quality basis sets), it is possible to carry out these calculations within a few days by using modern parallelizable algorithms.^22^

In a prior investigation of 4PN,^4^ we measured the CRD absorption spectrum of the T_1_(n,π*) ← S_0_ transition at room temperature. Most of the CRD assignments involved low-frequency, nontotally symmetric ring modes. We inferred their upper-level fundamental frequencies via observation of symmetry-allowed sequence bands such as *N*_1_^1^, considered along with known ground-state fundamentals. Now, under jet-cooled conditions, we observe several exceedingly weak, Franck–Condon forbidden, *N*_0_^1^ transitions that provide excited-state fundamentals more directly, without relying on combination differences involving ground-state vibrational levels. The ground-state frequencies are uncertain for some modes because they have only been measured in condensed-phase samples. Thus, detection of *N*_0_^1^ transitions in the present work has confirmed earlier sequence assignments and has improved the precision of the upper-state fundamentals for several nontotally symmetric modes.

In addition to this specific kind of improvement, the wavenumber assignments we present here are generally better in terms of accuracy and precision than in the room-temperature T_1_ ← S_0_ CRD spectrum because of the extreme narrowing of the rotational contours of the jet-cooled vibronic bands.

In the present study, we measured jet-cooled T_1_(n,π*) ← S_0_ vibronic transitions in a region up to about +900 cm^–1^ with respect to the origin band. In the room-temperature CRD spectrum, assignments in the higher wavenumber region are difficult, because T_1_ ← S_0_ vibronic features become submerged by hot bands associated with the S_1_(n,π*) ← S_0_ system. The latter has a 0_0_^0^ origin band that is about 1000 cm^–1^ above that of the T_1_ ← S_0_ system. At room temperature, the spin-allowed S_1_← S_0_ hot bands become much more intense than the spin-forbidden T_1_ ← S_0_ transitions, starting at about −400 cm^–1^ with respect to the S_1_← S_0_ origin, or +600 cm^–1^ relative to the T_1_ ← S_0_ origin band. Jet cooling suppresses the S_1_← S_0_ hot bands in this region, and has revealed previously indetectable T_1_ ← S_0_ bands.

With the experimental advances outlined above, we have been able to use spectroscopic measurements of 4PN to test T_1_(n,π*) frequency predictions of the EOM-EE-CCSD and TDDFT computational methods comprehensively. This analysis has revealed unexpected shortcomings of the more expensive EOM-EE-CCSD method in this application.

Finally, we provide insights from the analysis of the T_1_(n,π*) ← S_0_ origin band rotational contour of 4PN. The overall contour has a unique shape that is amenable to simulation, even though the resolution of the experiment (approximately 0.3 cm^–1^) does not permit assignment of individual rotational lines. We find that T_1_(n,π*) inertial constants obtained from both TDDFT and EOM-EE-CCSD calculations can produce acceptable band-contour simulations. However, agreement between simulated and observed contours requires careful choices of spin–spin and spin–rotation parameters^10^ for the T_1_(n,π*) upper state. We have narrowed down a range of spin constants that will qualitatively optimize the contour simulations, given plausible (*i*.e., TDDFT or EOM-EE-CCSD) inertial constants. For medium-sized molecules such as 4PN in the gas phase, the literature contains very few experimental determinations of triplet-state spin constants.^7,23^ Continued experimental investigations along these lines could stimulate the refinement of computational approaches for evaluating spin interactions in rotating molecules.

## Experimental and Computational Details

### Experiment

Spectra reported here were measured at the University of Wisconsin-Eau Claire (UW-Eau Claire) using the apparatus described below. At Purdue University, we conducted preliminary experiments to optimize jet expansion conditions and record initial spectra of the T_1_(n,π*) ← S_0_ band system of 4PN.

We conduct PE experiments at UW-Eau Claire Claire using a vacuum chamber consisting of a 6-way cross with ISO160 flanges and pumped by an Edwards Diffstak 100 diffusion pump.^24^ A pulsed nozzle (Parker Series 9), with an orifice of 1.0 mm, supplies gas for the free-jet expansion. The pressure of helium buffer gas in the stagnation chamber ranges from 1.0 to 5.0 atm (1.0 × 10^5^ to 5.0 × 10^5^ Pa), depending on the spectral features we are investigating. The pulsed nozzle operates at 10 Hz with a pulse width of about 220 μs. The jet expansion is directed toward the throat of the diffusion pump. Under these conditions, the time-averaged chamber pressure is below 1.0 × 10^–5^ Torr (1.3 × 10^–8^ Pa) when the nozzle is operating.

To record the spectra reported in this paper, we loaded typically 200 μL of liquid 4PN (Sigma-Aldrich) onto a glass-wool plug and located the plug in the pressurized helium tube leading to the pulsed nozzle. The temperature of the tube was maintained at about 90 °C with a resistive heating rope.

A tunable dye laser (Lambda-Physik Scanmate 2), equipped with a second harmonic generator (SHG), produces ultraviolet light for sample excitation. The dye laser is pumped by a frequency-doubled Nd:YAG laser (Continuum Surelite II), operating at 10 Hz. We used LDS 698 and LDS 722 dyes (Exciton) for the present experiments. The fundamental wavelength (vacuum) of the dye laser is calibrated to an uncertainty of ±0.002 nm using a wavemeter (Burleigh WA-4550). The nominal bandwidth of the dye-laser fundamental output is 0.1 cm^–1^ at 725 nm. The Nd:YAG pump laser is set to produce 150-mJ pulses at 532 nm, leading to maxiumum dye-laser output of about 30 mJ per pulse at 725 nm and 5 mJ/pulse of frequency-doubled (ultraviolet) light after the visible light passes through an angle-tuned β-(barium borate) SHG crystal.

To measure the T_1_(n,π*) ← S_0_ band system of 4PN, we attenuated the dye laser output to obtain ultraviolet pulse energies around 2.5 mJ throughout the interval between 353 and 367 nm. The ultraviolet laser beam passes through a biconvex lens with a focal length of 50 cm and then enters the vacuum chamber perpendicular to the jet expansion, so that the laser’s focal point is about 5 cm beyond the point where the laser and gas expansion cross. This crossing point is 13 mm (i.e., 13 nozzle diameters) downstream of the nozzle orifice.

After the laser excites the expanding gas sample, the isotropic emission is imaged onto a photomultiplier mounted outside the chamber and facing perpendicularly to the jet expansion. The apparatus for light collection includes a 2-in. (51 mm) diameter biconvex lens installed in an *f*/1 arrangement. The collection efficiency was improved by locating a spherical mirror (90 mm radius of curvature) across from the collection lens, so that rays emanating away from the collection lens reflect directly back onto themselves, enter the lens, and are focused onto the photomultiplier.

The present PE measurements of spin-forbidden T_1_(n,π*) ← S_0_ transitions require ca. 10 times greater laser fluence than typical fluorescence–excitation studies of spin-allowed transitions. To attenuate scattered laser light, a long-pass edge filter, with a cutoff wavelength of 375 nm, is located in front of the photomultiplier sensor. Even with the long-pass filter in place, the intense residual laser scatter tends to destabilize the photomultiplier, leading to artifacts in the emission decay curve at long times after the laser pulse. To eliminate this problem, we use a photomultiplier module equipped with a gating circuit (Hamamatsu H11706-40). The gate is kept closed during the laser pulse and opens 300 ns later. We record the photomultiplier signal over a time interval between 1.0 and 3.0 μs after the excitation. The phosphorescence decay signal drops to zero after approximately 8 μs. The decay function is a convolution of the long-lived radiative relaxation and the dropoff due to traversal of excited-state molecules out of the detector’s viewing region.

We record the phosphorescence decay curve by using a PC-based waveform digitizer (AlazarTech 9130) having 12 bits of vertical resolution and a 50 Ms/sec acquisition rate. At a given wavelength, we download and average the decay curves from typically 80 laser shots. From the averaged curve, we calculate and plot the average voltage over the selected time interval. We repeat this process at UV laser wavelength intervals of typically 0.01 nm to record an entire spectrum.

### Computational Methods

We carried out quantum-chemical calculations on the 4PN molecule as described previously.^4^ In brief, we used the Q-chem 5.2^25^ computational chemistry package for TDDFT calculations within the Tamm–Dancoff approximation. We performed a TDDFT geometry optimization of the T_1_(n,π*) state, followed by a harmonic-frequency calculation using the Perdew, Burke, and Enzerhof hybrid functional without the adjustable parameters (PBE0)^26^ XC functional and def2-TZVP basis set. We also used Q-chem 5.2 to carry out EOM-EE-CCSD geometry optimizations of the T_1_(n,π*) state, employing the cc-pVTZ, ANO1, or 6-311G(2pd,2df) basis set. The geometry optimizations were followed by EOM-EE-CCSD harmonic-frequency calculations using the CFOUR 2.1^27^ package. We used the frozen-core approximation for all EOM-EE-CCSD calculations.

We employed the WebMO graphical interface program^28^ to visualize and arrive at descriptions of the 4PN normal modes, and to render isosurfaces of the molecule’s canonical molecular orbitals.

## Results

### Vibronic Analysis

**Figure 2:**
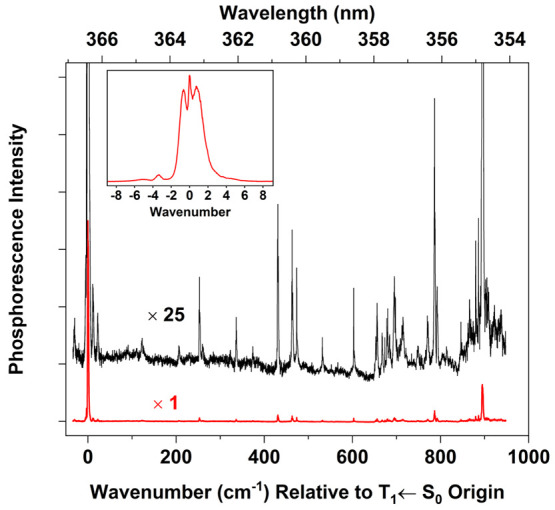
Overview of the T_1_(n,π*) ← S_0_ phosphorescence excitation spectrum of 4PN, recorded using a supersonic free-jet expansion. The buffer gas was 5 atm of helium. The maximum of the 0_0_^0^ origin band is located at 27293.2 cm^–1^. Inset shows the origin band on an expanded horizontal scale.

Figure 2 is an overview of the 4PN spectral region we measured via jet-cooled PE spectroscopy, using a helium backing pressure of 5 atm. We used rotational contours to confirm that the vibronic bands in this region belong to the T_1_(n,π*) ← S_0_ system. Later, we present a detailed analysis of the rotational structure; but in brief, the selection rules can be formulated using an angular-momentum coupling scheme analogous to Hund’s case (b) for linear molecules.^10,29^ A pattern-forming quantum number is *N*, which represents the rotation of the molecular framework in space. In 4PN, the T_1_(n,π*) ← S_0_ origin band shows a distinctive three-peak contour (Figure 2 inset), corresponding to intense *Q*-, R-, and *S*-form branches (*ΔN* = 0, +1, and +2, respectively) that are present within overlapping *ΔK*_a_ = 0 subbands.

Under the present jet-cooled conditions, the maximum of the 0_0_^0^ band is at 27 293.2 cm^–1^. This value is slightly different from the location of the origin-band maximum in the room-temperature CRD spectrum,^4^ 27 290.2 cm^–1^. In the latter case, the maximum occurs within the *Q*-form branch, whereas in the jet-cooled spectrum, the maximum is in the *R*-form branch.

Spin–orbit mixing between the T_1_(n,π*) (*A*_2_) state of 4PN and nearby singlet excited states provides the oscillator strength for the T_1_(n,π*) ← S_0_ electronic transition. The three-peak contour of the origin band is associated with a transition dipole moment that lies mainly in the *z* direction (Figure 1).^29^ This indicates that the dominant contribution to oscillator strength comes from the *S*_1_(π,π*) (*A*_1_) state.

**Figure 3:**
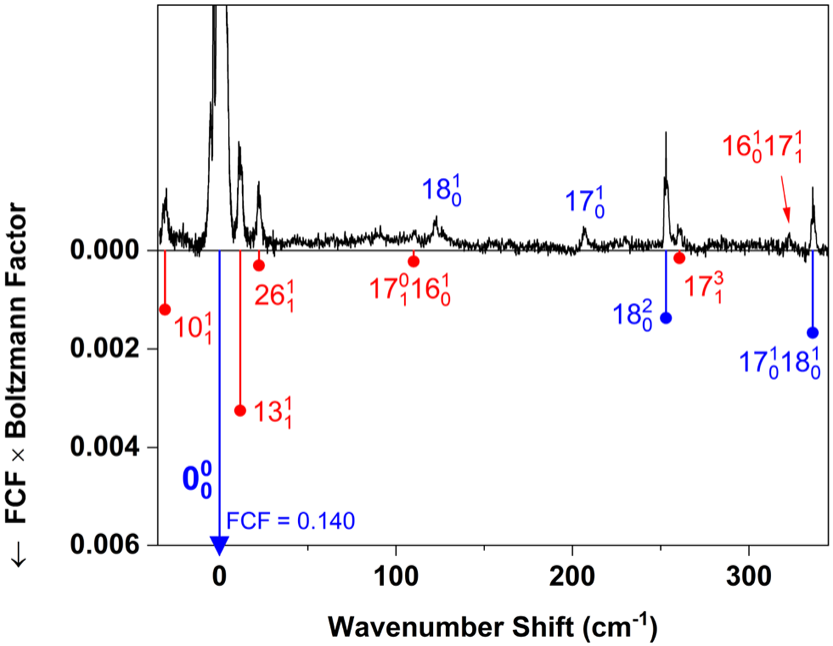
Low-wavenumber region of the spectrum in Figure 2. Blue tie lines show assignments of vibronic cold bands (*v*″ = 0), and red tie lies show assignments of hot bands (*v*″ > 0). Franck–Condon factors (FCF) were computed using a procedure described in the text. Assignment labels without tie lines, shown above the spectrum, refer to FC-forbidden bands.

Other assigned bands within the T_1_ ← S_0_ system have the same three-peak rotational contour as the origin band, as long as the vibrational wave functions in the ground and excited state belong to the same irreducible representation in the *C*_2*v*_ point group, so that the Franck–Condon (FC) factor is nonzero. In other cases (for example, the 18_0_^1^ (*b*_1_) out-of-plane ring-bending fundamental), the FC factor vanishes by symmetry, but the transition is made very slightly allowed through vibronic interaction between T_1_(n,π*) and another triplet excited state. (The electronic symmetry of the T_1_(n,π*) state is *A*_2_, and so the overall vibronic symmetry of a *b*_1_ vibrational state in T_1_ is (*b*_1_ × *A*_2_) = *B*_2_. These vibronic states can interact with the T_3_(π,π*) *B*_2_ electronic state, which itself gains oscillator strength via spin–orbit coupling with the S_2_(π,π*) *A*_1_ state. The spin–orbit coupling is mediated by the |*y*⟩ (*B*_2_) triplet spin component.) Fundamental bands in this category, such as 18_0_^1^ and 17_0_^1^ (*b*_1_ out-of-plane carbonyl wag), are extremely weak, but we predicted their locations within 1 cm^–1^ by using sequence band positions (e.g., 18_1_^1^) available from the room-temperature CRD spectrum,^4^ along with known^30^ ground-state fundamentals. Figure 3 shows the 18_0_^1^ and 17_0_^1^ band assignments in the jet-cooled spectrum.

**Figure 4:**
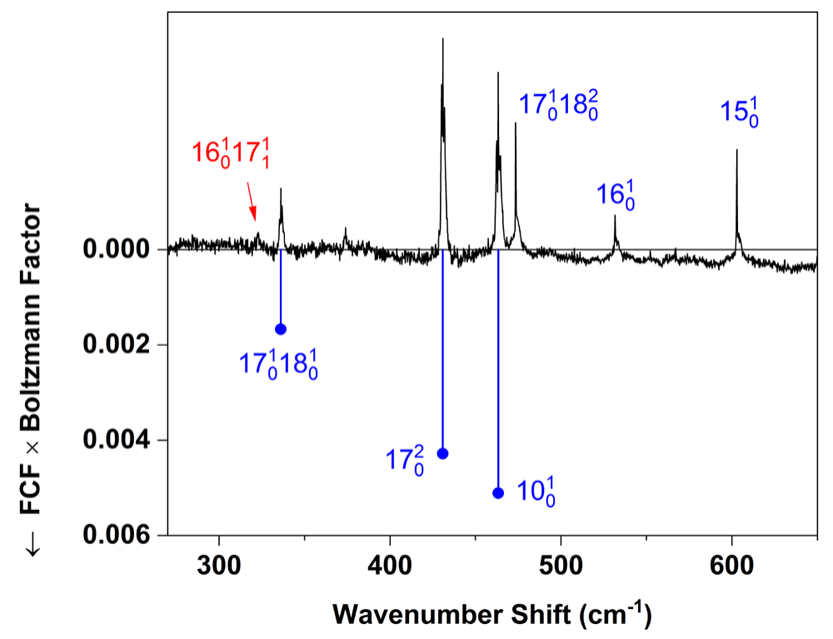
Continuation of the spectrum in Figure 3.

**Figure 5:**
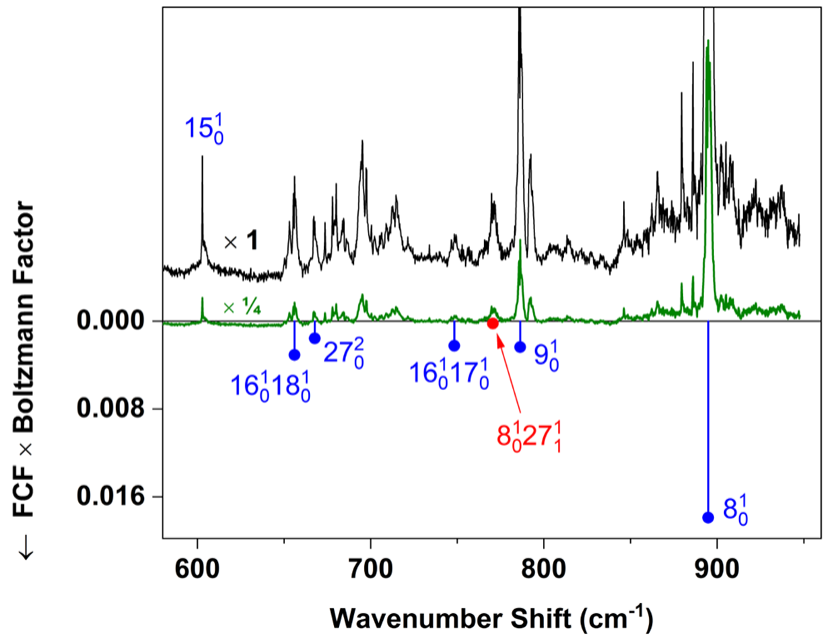
Continuation of the spectrum in Figure 4. The black trace is plotted using the same vertical scale as in Figures 3 and 4. The green trace is plotted on a contracted (× 1/4) vertical scale in order to show peaks near 800 and 900 cm^–1^, which have maximum intensity.

Higher-frequency band assignments, both FC-allowed and forbidden, are indicated in Figures 3, 4, and 5. To facilitate the assignments, we used a TDDFT (PBE0) harmonic-frequency calculation of the T_1_(n,π*) state, reported previously in conjunction with our room-temperature CRD investigation of 4PN.^4^ The TDDFT calculation provided normal-mode descriptions listed in Table 1.

**Table 1:** Calculated Normal Modes for the T_1_(n,π*) State of 4PN

Mode number	Symmetry	Description
27	*b*_2_	in-plane carbonyl wag
26	*b*_2_	in-plane ring bend
18	*b*_1_	out-of-plane ring bend
17	*b*_1_	out-of-plane carbonyl wag + ring bend
16	*b*_1_	ring inversion
15	*b*_1_	out-of-plane C–H wag + ring bend
13	*a*_2_	ring twist
10	*a*_1_	ring breathe
9	*a*_1_	ring breathe + carbonyl stretch
8	*a*_1_	ring breathe + C–O–C symmetric stretch

We used the normal-mode vectors from this calculation, along with normal modes of the ground state from a DFT (PBE0) calculation, to estimate the FC factors for vibronic bands in the T_1_(n,π*) ← S_0_ spectrum. We calculated the relative intensity of each band as (FC factor × Boltzmann factor). This product is represented in Figures 3–5 by the length of a tie line attached to an observed vibronic band. The Boltzmann factors were determined using a nominal vibrational temperature of 150 K, along with experimental^30^ ground-state vibrational frequencies. The entire observed spectrum was scaled vertically to make the origin-band maximum numerically equal to its calculated FC factor. Thus, for a given vibronic band, the tie line representing the calculated intensity (FC factor × Boltzmann factor) will match the peak height if the assignment is correct and the relative intensity is quantitatively accurate. This condition helped us finalize assignments of FC-allowed vibronic bands.

Intensity predictions are not readily available for FC-forbidden bands (those made allowed by triplet–triplet vibronic interaction). These bands have distinctive rotational contours containing a single narrow maximum. In these cases, for example the 15_0_^1^ and 16_0_^1^ fundamentals, assignments are based simply on proximity to the band positions predicted by the TDDFT calculation.

**Figure 6:**
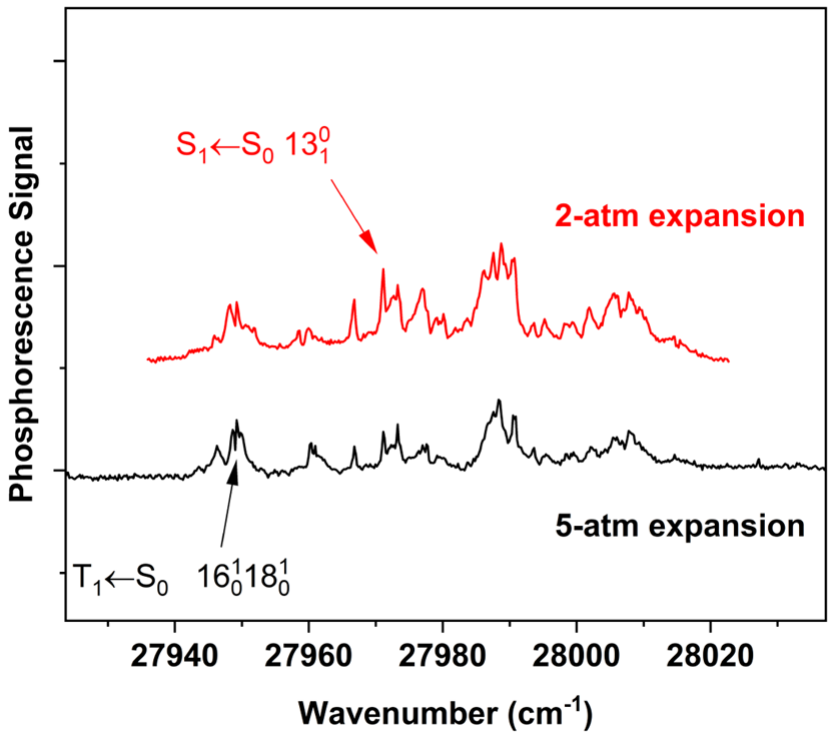
Region of T_1_(n,π*) ← S_0_ phosphorescence excitation spectrum of 4PN, about 700 cm^–1^ above the origin band. These traces show the suppression of S_1_(n,π*) ← S_0_ hot bands under colder helium expansion conditions.

In Figures 3–5, hot-band assignments are indicated in red. Hot-band sequences 10_1_^1^, 13_1_^1^, and 26_1_^1^ are prominent, despite the jet cooling. The FC factors for these bands are large and nearly the same as that of the origin band, because the ring geometry does not change significantly upon electronic excitation. In the highest-wavenumber region of the spectrum (Figure 5), the number of T_1_(n,π*) ← S_0_ assignments is limited because of overlapping hot bands belonging to the S_1_(n,π*) ← S_0_ system. Figure 6 shows the result of reducing the helium pressure to produce a warmer jet expansion in this region of the spectrum. The intensity of S_1_(n,π*) ← S_0_ vibronic hot bands increases under these conditions. An example is the peak at 27 971 cm^–1^, assigned to the 13_1_^0^ band of the S_1_← S_0_ transition. The origin of this band system^21^ is at 28 365 cm^–1^, and the −394 cm^–1^ shift corresponds to the experimentally known^30^ ground-state fundamental for ν_13_.

Table 2 lists the observed vibronic band positions, along with the assignments shown in Figures 3–5. The locations of combination bands and overtones are nearly equal to those inferred from observed fundamentals (see second column of Table 2), with discrepancies attributable to anharmonicity. Mode 17 (out-of-plane carbonyl wag) is particularly anharmonic, as we have discussed previously.^4^

**Table 2:** Vibronic Band Positions^a^ in the T_1_(n,π*) ← S_0_ PE Spectrum of Jet-Cooled 4PN

Observed pos. (cm^–1^)	Inferred pos. (cm^–1^)	Assignment	Inference (harmonic approx.) based on fundamental frequencies^b^ (cm^–1^)	Prev. assign.^c^
–30.3^d^	–43	10_1_^1^	463.3 – 504	10_1_^1^
–23.1	–25.8	18_1_^1^	123.0 – 148.76	18_1_^1^
0		0_0_^0^		0_0_^0^
11.7		13_1_^1^		
22.3		26_1_^1^		13_1_^1^
110.1	107.7	16_0_^1^17_1_^0^	531.6 – 423.92	16_0_^1^17_1_^0^
123.0		18_0_^1^		
206.2		17_0_^1^		
253.0	246.0	18_0_^2^	2(123.0)	
260.6	194.7	17_1_^3^	3(206.2) – 423.92	
322.4	313.9	16_0_^1^17_1_^1^	531.6 + 206.2 – 423.92	
336.1	329.2	17_0_^1^18_0_^1^	206.2 + 123.0	17_0_^1^18_0_^1^
430.9	412.4	17_0_^2^	2(206.2)	17_0_^2^
463.3		10_0_^1^		10_0_^1^
473.5	452.2	17_0_^1^ 18_0_^2^	206.2 + 2(123.0)	
531.6		16_0_^1^		
602.9		15_0_^1^		
656.0	654.6	16_0_^1^ 18_0_^1^	531.6 + 123.0	
667.7	662	27_0_^2^	2(331)^e^	
748.3	737.8	16_0_^1^ 17_0_^1^	531.6 + 206.2	
770.6		8_0_^1^ 27_1_^1^		8_0_^1^27_1_^1^
786.3		9_0_^1^		9_0_^1^
894.8		8_0_^1^		8_0_^1^

a Listed band positions are maxima relative to the 0_0_^0^ origin-band maximum, observed at 27 293.2 cm^–1^.

b Excited-state fundamentals are from *N*_0_^1^ band positions in this work; ground-state fundamentals are from refs (30) and (31).

c Previous assignments from ref (4) are listed for bands observed both at room temperature (ref (4)) and in the present jet-cooled spectrum.

d Uncertainty in a band maximum, due to noise at the tops of the peaks, is ±0.5 cm^–1^.

e The fundamental frequency of ν_27_ in the T_1_(n,π*) state was estimated by using the difference in 8_0_^1^27_1_^1^ and 8_0_^1^ band positions, along with the ν_27_ ground-state fundamental from ref (30).

The vibronic band assignments in this study of jet-cooled 4PN are a superset of those we offered for the room-temperature CRD spectrum.^4^ Many of the CRD assignments were tentative because T_1_ ← S_0_ peaks sat atop a broad, uneven baseline containing hot bands belonging to both T_1_ ← S_0_ and S_1_← S_0_ transitions. In the present jet-cooled work, all bands are observed as distinct peaks emerging from a nearly flat baseline. Table 2 shows that the assignments preferred in the present jet-cooled investigation match those of the room-temperature CRD spectrum, with the exception of just the 26_1_^1^ band. This band was misassigned^4^ as 13_1_^1^ in the room-temperature spectrum. We now find that the 13_1_^1^ band is located at a position close to the 0_0_^0^ band, where it would have been submerged at room temperature by the very intense and broad origin peak.

We used the spectroscopic assignments discussed above to ascertain fundamental vibrational frequencies for the T_1_(n,π*) state of 4PN. These results are listed in Table 3. For modes of *a*_1_ and *b*_1_ symmetry, the fundamental frequency in the T_1_(n,π*) state was taken as the difference between the locations of the *N*_0_^1^ and 0_0_^0^ band maxima. For these modes, experimental uncertainty in the T_1_(n,π*) frequency is less than 1 cm^–1^ and is due mainly to noise at the top of observed peaks in the PE spectrum. For modes of *a*_2_ and *b*_2_ symmetry, the *N*_0_^1^ bands were not detectable, but we inferred their positions by adding the experimentally known ground-state fundamental to the *N*_1_^1^ band position measured in the PE spectrum. For these modes, experimental uncertainty in the T_1_ fundamental is on the order of 5 cm^–1^, because the required ground-state frequencies are available^30^ only for condensed-phase rather than dilute gas samples.

**Table 3:** Vibrational Frequencies (cm^–**1**^) in the T_1_(n,π*) and S_0_ Electronic States of 4PN

		ν_18_ (*b*_1_) oo-plane ring bend	ν_17_ (*b*_1_) oo-plane C=O wag	ν_16_ (*b*_1_) ring in-version	ν_15_ (*b*_1_) oo-plane C–H wag	ν_13_ (*a*_2_) ring twist	ν_27_ (*b*_2_) in-plane C=O wag	ν_26_ (*b*_2_) in-plane ring bend	ν_10_ (*a*_1_) ring breathe	ν_9_ (*a*_1_) ring stretch	ν_8_ (*a*_1_) ring breathe
*T*_1_(n,π*)	TDPBE0/def2-TZVP	137	270	584	676	422	353	671	476	821	933
	EOM/cc-pVTZ^a^	70	131	548	720	521	347	667	471	803	928
	EOM/ANO1	73	137	553	721	515	349	668	471	803	926
	EOM/6-311G(2pd,2df)	106	155	560	728	—b	347	669	472	805	932
	**Expt. fundamental**^c^	**123.0**	**206.2**	**531.6**	**602.9**	**407**	**331**	**663**	**463.3**	**786.3**	**894.8**
											
*S*_0_	Expt. fundamental	148.76^d^	423.92^d^	720^e^	847^e^	395^f^	453^f^	641^f^	504^f^	822^f^	920.^e^

a EOM-EE-CCSD calculations employed the frozen-core approximation, a preferred choice when using the cc-pVTZ or ANO1 basis set. Computed EOM-EE-CCSD results differ from those reported previously^4^ because the earlier calculations correlated all electrons.

b Frequencies of *a*_2_ modes are not available because of a computed conical intersection that causes repulsion between the ^3^(n,π*) and ^3^(π,π*) states at a *C*_2_ (twisted) geometry.

c From jet-cooled phosphorescence excitation spectrum (this work).

d From gas-phase infrared spectrum.^31^

e From gas-phase infrared spectrum.^30^

f From Raman spectrum of molten sample.^30^

For comparison with experimental fundamentals in the T_1_(n,π*) state, Table 3 contains harmonic-frequency predictions obtained using TDDFT and EOM-EE-CCSD computational methods. For the present TDDFT calculation, we chose the PBE0 XC functional because of its documented successes^32−34^ in predicting excited-state properties of carbonyl-containing molecules. The PBE0 functional also slightly outperformed the widely used B3LYP functional in our earlier examination^4^ of T_1_(n,π*) and S_1_(n,π*) vibrational frequencies of 4PN. The present investigation is similar to our previous^4^ computational study of 4PN, but we now employ basis sets better suited to the computational methods. For example, we previously carried out the TDDFT calculations using the cc-pVTZ basis set. However, the Dunning cc basis sets were designed for use with the frozen-core approximation, whereas DFT methods correlate all electrons. Thus, for the TDDFT calculations in the present work, we employed def2-TVZP, which is an all-electron basis set and contains polarization functions optimized^35^ for DFT calculations.

Unlike TDDFT calculations, the EOM-EE-CCSD (*ab initio*) frequency calculations reported in Table 3 do incorporate the frozen-core approximation, a conventional choice for post-Hartree–Fock methods. For these calculations, we used several different triple-ζ basis sets, all of the same size, that are compatible with a frozen core. In the Discussion section, we assess the relative performance of TDDFT (PBE0) vs EOM-EE-CCSD in predicting T_1_(n,π*) vibrational frequencies of 4PN.

### Rotational Analysis of the 0_0_^0^ Band Contour

**Figure 7:**
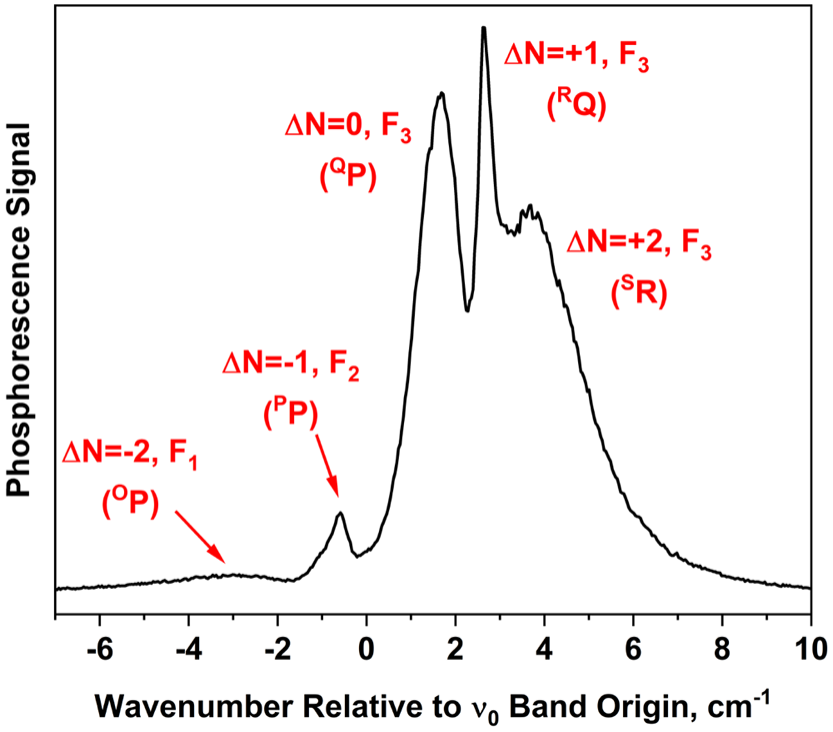
Jet-cooled T_1_(n,π*) ← S_0_ phosphorescence excitation spectrum of 4PN, recorded using a helium backing pressure of 2 atm. The region of the 0_0_^0^ band is shown. The band origin, listed in Table 4, was determined using a simulation procedure outlined in the text. Branch labels are given in the format ^*ΔN*^*ΔJ*, where the *O*-form branch corresponds to *ΔN* = −2; the *P*-form branch corresponds to *ΔN* = −1, etc.

Figure 7 shows the 0_0_^0^ band of the T_1_(n,π*) ← S_0_ transition of 4PN, recorded using a helium backing pressure of 2 atm. The wavelength increment between data points in the scan is 0.0005 nm, corresponding to about 0.04 cm^–1^ in this region of the spectrum. The wavenumber scale on the spectrum is relative to the band origin (ν_0_), determined to be 27 290.6 cm^–1^ using a simulation procedure described later in this section. The simulation also provided rotational branch labeling shown in the figure.

Our analysis of the band contour employs an angular-momentum coupling scheme analogous to Hund’s case (b) for diatomic molecules.^10,29^ This limiting case applies to triplet states of most organic molecules lacking heavy atoms. For these species, spin–orbit interactions with nearby electronic states are weak, and the electronic spin **S** is not coupled electrostatically to the nuclear framework. Thus, S is a good quantum number, but its projection along a molecular axis is not. Another good quantum number in the case (b) limit is *N*, which represents the rotation of the nuclear framework in space. The projection of **N** on the *a*-axis of the 4PN molecule is represented by *K*_*a*_, which has the same meaning as for a near-prolate asymmetric top molecule lacking spin. The total angular momentum **J** = **N** + **S** is conserved, so that for a triplet species (S = 1), the quantum number *J* can have the values *N* – 1, *N*, and *N* + 1. The corresponding rotational sublevels have the labels *F*_3_, *F*_2_, and *F*_1_, respectively. The labeled features in Figure 7 specify ^*ΔN*^*ΔJ* branches that are relatively intense according to selection rules.^29,36^ Each branch is the superposition of *ΔK*_*a*_ = 0, *K*″ = 0, 1, 2... sub-branches.

We employed the STROTA computer program, written by Judge et al.,^29^ to ascertain the branch labels in Figure 7. STROTA is capable of fitting or predicting the rotational structure of singlet–triplet spectra, irrespective of the chosen angular-momentum coupling scheme.

The dye-laser system in the present experiment does not permit resolution of individual rotational lines, and hence it is not possible to obtain a quantitative fit to the spectrum. However, we have been able to extract reasonable values of T_1_(n,π*) spin constants from the spectrum by using the simulation feature of the STROTA program. We fixed the T_1_(n,π*) inertial constants (*A*', *B*', and *C*') at values obtained in the TDDFT (PBE0) calculation outlined in the previous section. We used ground-state inertial constants known from microwave experiments.^37^ We varied other molecular parameters manually, including spin constants^10^*a*, *a*_0_, α, and β, and ran the STROTA program in simulation mode to check the quality of the fit. We also varied the band origin ν_0_, effective rotational temperatures characterizing the jet expansion, and transition dipole moment components. The inertial constants were kept fixed at the values referred to above.

**Figure 8:**
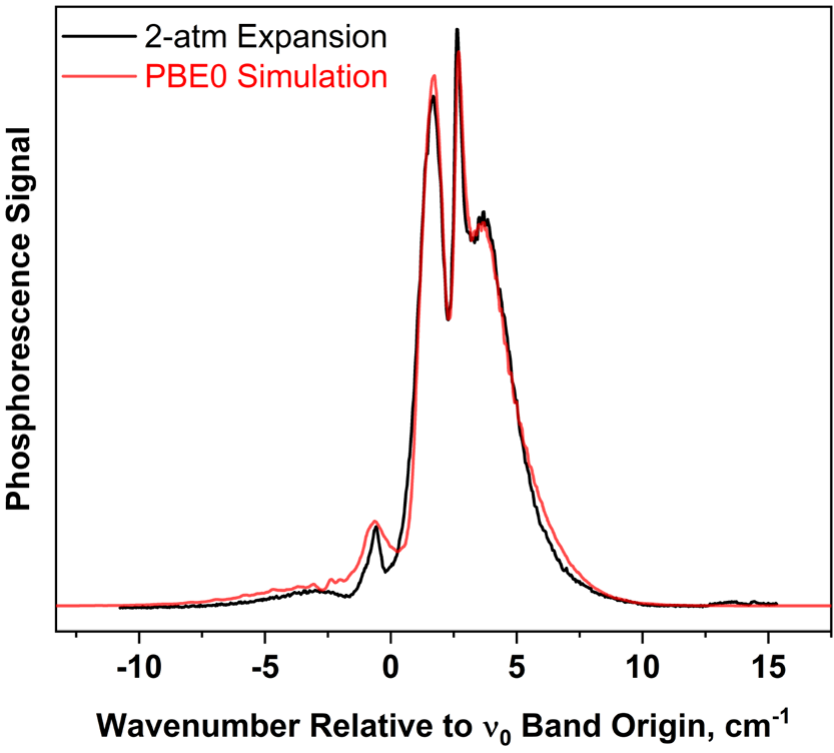
Experimental spectrum of Figure 7 (black trace), along with simulation (red trace) produced using the molecular constants listed in Table 4. The simulation used a two-temperature model (see text) to describe the distribution of rotational levels in the ground state. The two temperatures were chosen to be 4 and 16 K, with weighting factors of 0.4 and 0.6, respectively.

The band contour was simulated by convoluting the predicted rotational lines with a Gaussian function corresponding to the bandwidth of the doubled dye-laser output. We changed the variable parameters interactively until we achieved optimal qualitative agreement between the simulated and observed band contour. The final simulation, shown in Figure 8, was produced using molecular parameters listed in Table 4. The ratio of transition dipole moment components, μ_*x*_:μ_*z*_, was chosen to be 0.2:1, which reflects contributions to oscillator strength from ^1^(n,π*) *B*_1_ and ^1^(π,π*) *A*_1_ states, respectively.

**Table 4:** Molecular Constants (cm^–1^) Used To Simulate the T_1_(n,π*) ← S_0_ 0_0_^0^ Contour of 4PN

Ground-state inertial constants^a^	
*A*″	0.19544
*B*″	0.090566
*C*″	0.061895

Excited-state inertial constants^b^	
*A*′	0.19789
*B*′	0.089883
*C*′	0.061809

Spin constants	
*a*	0.1468
*a*_0_	0.1247
α	–1.245
β	–0.56

Band origin	
ν_0_	27290.6

a From the microwave spectrum.^37^

b From the TDDFT (PBE0)/def2-TZVP calculation reported in this work.

The simulation reproduces the locations of the band maxima well, though intensities are slightly inaccurate, particularly in the wavenumber extremes of the branches. This inaccuracy is due, in part, to the model we used to describe the rotational temperature of 4PN in the free-jet expansion. This experiment does not incorporate a skimmer, and therefore, the laser excites a collection of molecules located over a relatively large range of angle (θ) with respect to the centerline of the expansion. We estimate that the light-collection system in this experiment images phosphorescence from excited-state molecules spanning an angular range of θ = ±25°. The density of buffer gas is dependent on cos^2^(*f*·θ), where *f* is a numerical factor that depends on the heat-capacity ratio of the buffer gas. At |θ| = 25°, the density of the helium buffer gas is reduced by a factor of 0.77 compared to the centerline of the expansion.^38^ Thus, the equilibrium rotational temperature of the 4PN molecules could vary considerably over the sampled range.

The simulation feature of the STROTA program incorporates a single Boltzmann factor corresponding to rotational temperature. To account for temperature variation across the sampled region of the jet, we simply used a weighted sum of two spectra simulated at different rotational temperatures. We generated the optimized simulation in Figure 8 by choosing the two temperatures to be 4 and 16 K, with weighting factors of 0.4 and 0.6, respectively.

The two-temperature approach is known to be successful for a skimmed molecular beam,^39^ though for the present free expansion, the model is likely oversimplified, and this contributes to intensity deviations in the overall simulation. Nonetheless, these deviations are relatively small and are not significantly worse for slightly colder or warmer expansions. The Supporting Information contains band contours measured using 3-atm and 1-atm expansions of helium. Simulations using the two-temperature model (with the same molecular parameters as in Table 4) are also included. At the highest backing pressure used in this investigation (5 atm, inset of Figure 2), the two-temperature model is unable to reproduce the observed intensity profile suitably.

**Figure 9:**
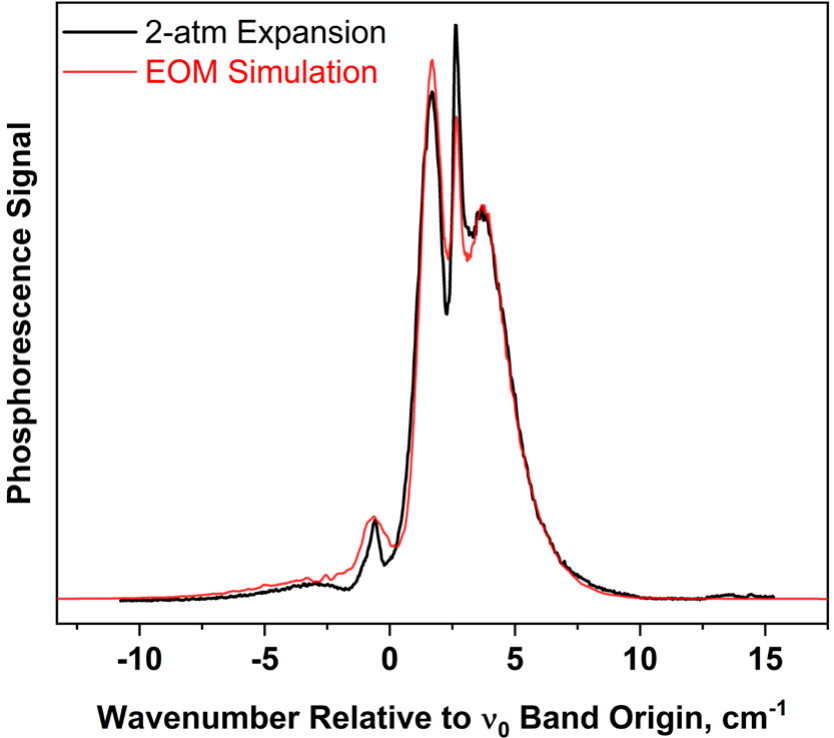
Experimental spectrum of Figure 7 (black trace), along with simulation (red trace) produced using T_1_(n,π*) inertial constants from an EOM-EE-CCSD calculation rather than TDDFT (PBE0).

A caveat of the overall rotational analysis presented above is that the spin-rotation constants (*a* and *a*_0_) and the inertial constants both depend on the molecule’s moments of inertia, and hence the two sets of constants could be correlated with each other. We investigated this premise by using T_1_(n,π*) inertial constants from an EOM-EE-CCSD calculation discussed earlier, rather than the TDDFT (PBE0) calculation, in the band-contour simulation. The inertial constants from the EOM-EE-CCSD/cc-pVTZ calculation are (in cm^–1^) *A*′ = 0.19502; *B*′ = 0.089349; and *C*′ = 0.061276. We used the EOM-EE-CCSD inertial constants to simulate the band contour observed in the 2-atm helium expansion. The result is shown in Figure 9.

In generating this simulation, we kept the spin–spin constants (α and β) and the band origin (ν_0_) the same as those optimized previously in conjunction with the TDDFT calculation of inertial constants. However, because of the correlation between inertial and spin-rotation constants, we needed to adjust the latter in order to achieve a qualitatively reasonable fit when using EOM-EE-CCSD inertial constants. The spin-rotation constants used in the simulation of Figure 9 are *a* = 0.1559 cm^–1^ and *a*_0_ = 0.1392 cm^–1^. These values are about 10% higher than those used with the TDDFT inertial constants, an outcome that places a rough uncertainty range on the spin-rotation constants available from the observed band contour.

In contrast to the spin–rotation constants, the spin–spin constants, α and β, do not involve the molecular moments of inertia directly. The spin–spin constants are related to the average positions of the unpaired electrons with respect to the molecular framework and can also include spin–orbit contributions.^10^ In the triplet state of a near-symmetric rotor, the value of α controls the splitting among *F*_1_, *F*_2_, and *F*_3_ sublevels for a given (*N*,*K*). In the case of 4PN, the magnitude of α is large enough that three separate T_1_(n,π*) ← S_0_ subbands, corresponding to the three *F* sublevels, are resolvable within the 0_0_^0^ band contour. The intensity within a given *F* subband depends sensitively on the *ΔN* branch,^29^ but the most intense branches are readily differentiated from each other, as seen in Figure 7.

**Figure 10:**
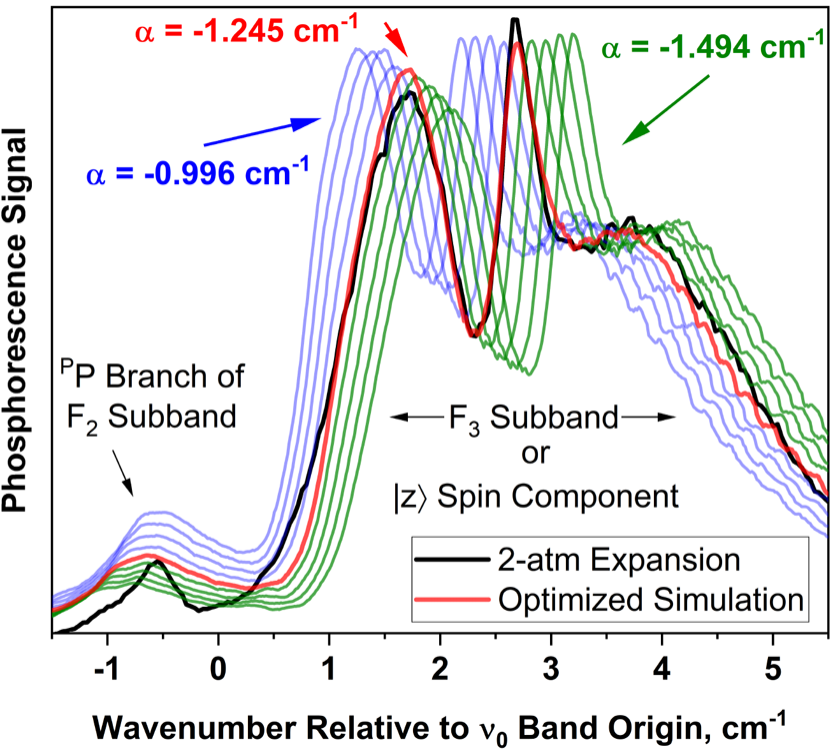
Origin band of the 4PN T_1_(n,π*) ← S_0_ transition, simulated using a range of α values departing from its optimum (red trace) by +20% to −20%. Other molecular constants are those in Table 4. Simulations employing positive and negative deviations in α are shown in blue and green, respectively. Black trace is the experimental spectrum.

We confirmed the role of α described above by generating simulations of the 0_0_^0^ band using a range of α values that depart from its optimum (−1.245 cm^–1^) by ±20%. These simulations are shown, along with the observed band contour for the 2-atm expansion, in Figure 10. In the simulations, the *Q*-, *R*-, and *S*-form branches (*ΔN* = 0, +1, and +2, respectively) shift together as α is varied, because these intense branches belong to the same *F*_3_ subband (see Figure 7 for branch labels). The *P*-form branch shifts minimally because it is part of the *F*_2_ subband, whose position depends less sensitively^10^ on α.

The optimized simulation in Figure 10 reproduces the separation between *F*_2_ and *F*_3_ subbands very well, indicating that the parameter value α = −1.245 cm^–1^ is accurate. When α is varied by ±20% of its optimum, the shift in the *F*_3_ subband results in an extreme departure from the observed spectrum and suggests that the uncertainty in α is less than about 10%.

The spin–spin constant β is an asymmetry parameter that does not affect the triplet rotational energies unless *K*_*a*_ = 1.^10^ In the observed jet-cooled spectrum of 4PN, triplet levels up to *K*_*a*_ = 5 make significant contributions, and therefore the optimal value of β cannot be determined precisely at the resolution of this experiment. We find that values within ±50% of β = −0.56 cm^–1^ produce acceptable simulations.

## Discussion

This investigation provides new details about the structure and dynamics of 4PN in its T_1_(n,π*) state. The jet-cooled T_1_(n,π*) ← S_0_ spectrum precisely and unambiguously gives fundamental frequencies for the lowest-energy modes of the molecule, ν_15_ through ν_18_. We determined these frequencies directly, through measurement of *N*_0_^1^ bands. The very weak (Franck–Condon-forbidden) *N*_0_^1^ bands for out-of-plane modes had not been detectable in a previous room-temperature investigation^4^ because of spectral congestion.

Experimental fundamentals (Table 3) for the T_1_(n,π*) ring modes and higher-frequency vibrations allow a comprehensive assessment of TDDFT (PBE0) vs EOM-EE-CCSD performance, presented below. This analysis also reflects basis-set choices that are better suited to the computational methods than in our previous study.^4^

**Figure 11:**
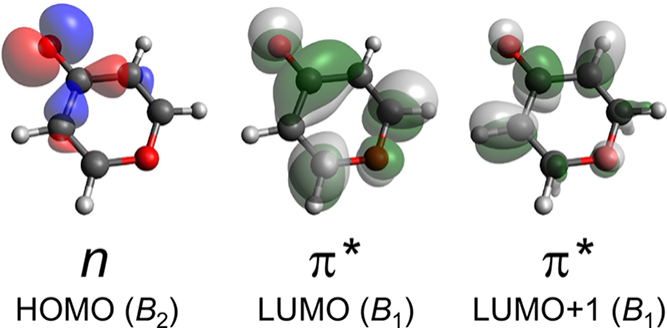
Isosurfaces of the canonical molecular orbitals for the lowest-energy electronic transition of 4PN. The value of |ψ| on each isosurface is 0.050 Å^–3/2^.

Success of these excited-state frequency calculations hinges on the method’s ability to characterize the π*← *n* chromophore in 4PN. Figure 11 shows the canonical (Hartree–Fock) HOMO–LUMO pair. Occupancy of the π* LUMO not only weakens the carbonyl bond but also affects the bonds within the ring, in accordance with this orbital’s nodal structure. The most direct consequence of this delocalization is frequency lowering of most of the ring vibrational modes upon T_1_(n,π*) ← S_0_ excitation.

For the in-plane modes (*a*_1_ and *b*_2_ symmetry) listed in Table 3, the TDDFT and EOM calculations of the *T*_1_(n,π*) state have about the same high accuracy, producing harmonic-frequency values that are greater than the experimental fundamentals by a just a few percent or less. Overestimates of this magnitude can be attributed to neglect of anharmonicity.

For out-of-plane modes (*a*_2_ and *b*_1_ symmetry), computed frequencies are generally much less accurate, with errors in both directions that vary considerably among computational method, basis set, and mode number. We can understand these errors on a case-by-case basis.

The ν_18_ (*b*_1_) mode is the lowest-frequency vibration in the 4PN molecule. This normal mode is an out-of-plane ring-bending motion in which the *a* axis acts as a hinge. The ν_18_ mode also involves significant torsion about each of the C–C single bonds flanking the carbonyl group. The torsional motion becomes less stiff upon T_1_(n,π*) ← S_0_ excitation because electron promotion to the LUMO (Figure 11) disrupts O=C–C=C conjugation. The observed ν_18_ fundamental frequency drops from 149 cm^–1^ in the ground state to 123 cm^–1^ upon excitation to *T*_1_(n,π*). Table 3 shows that the EOM calculation significantly overestimates this drop, and the TDDFT calculation modestly underestimates it.

We investigated these errors with the aid of the Natural Bond Orbital (NBO)^40^ program. We carried out an NBO analysis of the T_1_(n,π*) electron density obtained in the EOM and TDDFT calculations. According to the TDDFT calculation, T_1_(n,π*) ← S_0_ excitation promotes effectively 0.999 electron to the LUMO of Figure 11. The EOM calculation predicts an occupancy of 0.972 electron for this LUMO. In the EOM calculation, 0.011 electron is associated with the HOMO →LUMO+1 promotion. Compared to the LUMO, the electron density in the LUMO+1 is more localized to the region of the carbonyl moiety, and the LUMO+1 has nodes at the C–C bonds adjacent to the carbonyl. These characteristics lower the resistance to torsion about these bonds and help to explain why the EOM calculation produces a ν_18_ frequency significantly lower than the observed fundamental.

The ν_17_ (*b*_1_) mode is a ring-bending vibration similar to ν_18_, except ν_17_ involves out-of-plane carbonyl wagging (*i*.e., pyramidalization) in addition to ring bending. As shown in Table 3, the EOM calculation of T_1_(n,π*) seriously underpredicts the ν_17_ harmonic frequency. In this case, as with ν_18_, the EOM calculation appears to overestimate the contribution of the LUMO+1 (Figure 11). The nodal structure of this orbital diminishes repulsion between the C–C bonds adjacent to the carbonyl. The consequence for ν_17_ is that the carbonyl carbon is subjected to unrealistically low resistance to pyrimidalization in the EOM calculation. On the other hand, it is possible that the TDDFT calculation lacks sufficient LUMO+1 character, leading to a significant overestimate of the ν_17_ frequency.

Other observed modes are more delocalized to include atoms distant from the carbonyl group, and it is not possible to analyze their calculated frequencies in terms of the simple HOMO–LUMO picture presented above. For example, the calculation of ν_13_ (*a*_2_) is influenced by configuration interaction in a subtle way. The ν_13_ vibration takes the molecule from its *C*_2*v*_ equilibrium structure to a ring-twisted geometry of *C*_2_ symmetry. For this mode, the TDDFT harmonic-frequency prediction is very accurate, producing a ν_13_ value that is just 4% greater than the observed T_1_(n,π*) fundamental of 407 cm^–1^. However, the EOM calculation of this mode is pathological, with results sensitive to the basis set. The correlation consistent ANO1 and cc-pVTZ basis sets lead to a 28% overestimate of ν_13_, whereas with the 6-311G(2pd,2df) basis, the EOM calculation produces an imaginary frequency.

We have traced^4^ these problems to a computed conical intersection between the T(n,π*) and T(π,π*) surfaces. The intersection occurs at a *C*_2*v*_ geometry, where the two electronic states are classified as ^3^*A*_2_ and ^3^*A*_1_, respectively. The T(n,π*) state has a nearby equilibrium geometry, also *C*_2*v*_, and at this point in coordinate space, the ^3^*A*_2_ and ^3^*A*_1_ states remain close to each other energetically, but they do not interact because they still have different electronic symmetry. However, at ring-twisted (*C*_2_) geometries associated the with ν_13_ coordinate, the T(n,π*) and T(π,π*) states have the same symmetry and can interact with each other. The EOM calculation finds the two states to be nearly isoenergetic at the T(n,π*) minimum, and thus they mix significantly along the ν_13_ coordinate. With the ANO1 and cc-pVTZ basis sets, the T(π,π*) state is located slightly below T(n,π*), and the mixing of the two states pushes T(n,π*) upward along the ν_13_ coordinate. This leads to overestimation of the ν_13_ frequency. However, the 6-311G(2pd,2df) basis set locates T(π,π*) slightly *above* T(n,π*), so that the latter is repelled downward, giving the surface a downward curvature and an imaginary frequency.

By contrast to EOM, the TDDFT calculation locates the T(π,π*) state *s*ignificantly higher than T(n,π*) in this potential-energy region. The TDDFT calculation predicts a T_2_(π,π*) – T_1_(n,π*) energy difference of about +0.16 eV, or 1300 cm^–1^, at the T_1_(n,π*) minimum. The larger state separation allows the TDDFT calculation to produce a highly accurate ν_13_ frequency for T_1_(n,π*); *i*.e., 422 cm^–1^, compared to the measured value of 407 cm^–1^.

Aside from these unique findings for ν_13_, our conclusion is that the EOM calculation tends to underestimate the frequencies of out-of-plane modes in the T_1_(n,π*) state of 4PN, whereas the TDDFT calculation tends to overestimate them slightly. The EOM description appears to contain unduly large contributions from molecular orbitals like the LUMO+1 shown in Figure 11. Such orbitals have more extensive antibonding character at the carbonyl than does the LUMO.

These considerations help to interpret the T_1_(n,π*) equilibrium geometries computed by TDDFT and EOM, as well as the quality of 0_0_^0^ band contour simulations. As seen in the contour analysis (Results section), the TDDFT and EOM calculations do not differ significantly in their prediction of the *B*′ or *C*′ inertial constant for the T_1_(n,π*) state. However, the predicted *A*′ constant from the TDDFT calculation is slightly greater than that of EOM, with values of 0.1978 and 0.1950 cm^–1^, respectively. For comparison, the experimentally measured *A*″ constant for the ground state is 0.1954 cm^–1^.^37^ Thus, the TDDFT calculation, but not EOM, predicts a contraction of the ring structure toward the *a* axis upon T_1_(n,π*) ← S_0_ excitation. The contraction predicted by TDDFT can be traced, in part, to a pronounced shortening of the two C–C bonds flanking the carbonyl group. The TDDFT calculation predicts a length of 1.418 Å for these bonds, whereas EOM predicts 1.436 Å.

Irrespective of the particular computational method, the π* ← n promotion should cause shortening of these C–C bonds, in accord with the LUMO isosurface shown in Figure 11. The TDDFT description of the T_1_(n,π*) ← S_0_ excitation does not include contributions from the LUMO+1 or higher molecular orbitals, according to the NBO analysis discussed above. This helps explain why the TDDFT calculation predicts a more extreme shortening of the C–C bonds adjacent to the carbonyl, compared to the EOM calculation. The latter explicitly includes contributions from orbitals such as LUMO+1, which have nodes at these C–C bonds.

As seen in Figure 8, the simulated 0_0_^0^ band contour arising from the TDDFT (PBE0) inertial constants agrees well with the observed spectrum. The agreement is slightly better for the TDDFT structure than for EOM, as seen by comparison to Figure 9. The larger *A*′ constant from the TDDFT calculation subtly affects the branch structure (labeled in Figure 7) and leads to the better agreement. Hence, it appears that the TDDFT calculation is more accurate than EOM in predicting the ring bond lengths of the *T*_1_(n,π*) excited state.

We conclude this section by noting a surprising outcome of the 0_0_^0^ band contour analysis. The spin constants determined for the T_1_(n,π*) state of 4PN (Table 4) are an order of magnitude greater than is expected for monocyclic organic molecules.^7,9,10,41^ For example, the value of α in the T_1_(n,π*) state of 2-cyclopenten-1-one (2CP) is −0.25 cm^–1^,^7^ in contrast to −1.25 cm^–1^ determined for 4PN in the present study. In the triplet states of molecules without significant spin–orbit coupling, the α constant is proportional to ⟨(1–3 cos θ)/*r*^3^⟩,^41^ where *r* is the distance between the unpaired electrons, and θ describes the orientation of the spin–spin vector in the molecular frame. (The negative sign of α in 4PN indicates that the distribution of spin density is prolate.^41^) Given the similarity of 2CP and 4PN, in terms of their size, shape, and π* ← *n* chromophore, one expects similar α constants for their T_1_(n,π*) states. But the experimental α values are significantly different in the two molecules, and the magnitude of α for 4PN is closer to that of O_2_ (1.32 cm^–1^^42^) in its triplet ground state.

In triplet states of O_2_ and isoelectronic diatomics, spin–orbit interaction makes a dominant contribution to α,^43^ and the angular momentum coupling is close to the Hund’s case (a) limit. In the T_1_(n,π*) state of 4PN, the spin–orbit energy should be negligible because the orbital angular momentum averages out to zero in nonlinear polyatomic molecules. Nonetheless, the value of α in the T_1_(n,π*) state of 4PN is large enough to split the *F* spin-rotation components by an amount greater than the rotational intervals—indicating that the angular momentum coupling has departed from the Hund’s case (b) limit and begins to resemble that of case (a).

As the case (a) limit is approached, the |*z*⟩ triplet spin substate (body-fixed axis) becomes relatively uncontaminated by |*x*⟩ and |*y*⟩, and the projection of electron spin on the molecular symmetry axis becomes a good quantum number. At the same time, the *N* quantum number becomes less useful for describing the rotational level pattern and is replaced by *J*. In the singlet–triplet spectra of case (a) molecules having *C*_2*v*_ symmetry, each triplet spin state (|*x*⟩, |*y*⟩, and |*z*⟩) is represented as a separate band having the characteristic *P*, *Q*, *R* branch structure (referring to *ΔJ*) of a singlet–singlet transition.^29^ The branch labels in Figures 7 and 10 show that this behavior is manifested in the *F*_3_ subband of the 4PN T_1_(n,π*) ← *S*_0_ transition. This appears to be an example of Hund’s case (ab) triplet-state coupling,^44^ in which one of the three *F* components of the case (b) representation is sufficiently distant from the other two that case (a) labeling becomes meaningful for the unique component. In the T_1_(n,π*) ← S_0_ transition of 4PN, the *F*_3_ component is aptly described as a |*z*⟩ subband.^29^

To be sure, it is unusual for triplet states of an asymmetric-top molecule to fall into the case (a) or (ab) category, because the first-order contribution to spin–orbit coupling is zero. However, second-order spin–orbit interaction, which couples two electronic states, can be highly significant—particularly if heavy atoms are involved. In deuterated selenoformaldehyde (D_2_C^80^Se), for example, the T_2_(π,π*) state interacts particularly strongly with the |*x*⟩ and |*y*⟩ components of T_1_(n,π*).^45^ In the 4_0_^1^ band of the selenoformaldehyde-*d*_2_ T_1_(n,π*) ← S_0_ system, this spin–orbit interaction leads to a |*z*⟩ subband that is separated from the other two by more than 100 cm^–1^—a definitive example^46^ of case (ab) coupling.

In the T_1_(n,π*) state of 4PN, the allowed spin–orbit interactions with other electronic states are isomorphic with those of selenoformaldehyde, as both the 4PN and selenoformaldehyde T_1_(n,π*) states have *A*_2_ orbital symmetry. In 4PN, the magnitude of second-order spin–orbit matrix elements must be considerably smaller than in selenoformaldehyde, because the latter has the “heavy-atom” effect. However, the energy gap between T_2_(π,π*) and T_1_(n,π*) is likely much smaller in 4PN than in selenoformaldehyde. Our TDDFT calculation of 4PN predicts a T_2_ – T_1_ energy difference of 1300 cm^–1^ near the T_1_(n,π*) minimum, whereas this difference is calculated^45^ to be about 5000 cm^–1^ in selenoformaldehyde. Because of the relatively small T_2_ – T_1_ energy gap in 4PN, it is entirely plausible that second-order spin–orbit perturbations are responsible for the splittings, on the order of a few cm^–1^, observed among the T_1_(n,π*) spin states in the T_1_(n,π*) ← S_0_ spectrum of 4PN.

## Conclusions

The 4PN molecule provides an excellent testbed for understanding structural effects of electronic excitation. The canonical HOMO–LUMO picture of π*← *n* excitation is straightforward and leads to qualitatively predictable changes in vibrational frequencies. It should be possible to predict these changes with quantitative accuracy by using modern computational approaches for treating excited states. To test these expectations, we have measured the vibronically resolved T_1_(n,π*) ← S_0_ band system of 4PN under jet-cooled conditions. We observe significant frequency decreases for out-of-plane ring modes upon excitation. These changes stem from the nodal structure of the π* LUMO. However, configuration interaction within the triplet manifold can lead to subtleties that are difficult to model accurately, even with high-level computational methods. The computationally intensive EOM-EE-CCSD technique tends to overestimate frequency drops for the out-of-plane modes. The much more economical TDDFT method modestly underestimates the drops but generally performs impressively with regard to frequency predictions. Also, the TDDFT (PBE0) calculation of T_1_(n,π*) inertial constants leads to very good agreement between simulated and observed rotational contours for the T_1_(n,π*) ← S_0_ 0_0_^0^ origin band.

Both computational methods reveal that the T_2_(π,π*) state is nearly isoenergetic with T_1_(n,π*) at molecular geometries close to the T_1_(n,π*) potential-energy minimum. The EOM calculation places the two states too close together, leading to unphysical predictions of the lowest ring-twisting frequency. However, the TDDFT (PBE0) calculation locates the T_2_(π,π*) at an energy that leads to a very accurate ring-twist frequency. Moreover, the TDDFT energies support the hypothesis that the T_1_(n,π*) and T_2_(π,π*) are coupled via spin–orbit interaction. A spin–orbit perturbation of this sort could explain the signficant departure from the expected Hund’s case (b) branch structure we observe in the T_1_(n,π*) ← *S*_0_ 0_0_^0^ band. We hope this hypothesis promotes expansion of computational tools for investigating triplet states.

## Supporting Information Available

This document shows origin-band contours for the T_1_(n,π*) ← S_0_ transition of 4PN, measured using 3-atm and 1-atm expansions of helium. Simulations using the two-temperature model (with the same molecular parameters as in Table 4) are also included. (PDF)
