# Supplementary material for: Jet-Cooled Phosphorescence Excitation Spectrum of the T1(n,π*) ← S0 Transition of 4H-Pyran-4-one
Source: J Phys Chem A. 2023 Apr 17;127(16):3636–47. doi: 10.1021/acs.jpca.3c01059 (PMC10150392; doi:10.1021/acs.jpca.3c01059)
Supplement: Supplementary file 1 — jp3c01059_si_001.pdf [file jp3c01059_si_001.pdf]

Jet-Cooled Phosphorescence Excitation Spectrum of the $T_1(n,\pi^*) \leftarrow S_0$ Transition of 4*H*-Pyran-4-one

Sean W. Parsons,[†,‡] Devon G. Hucek,[†,¶] Piyush Mishra,[§,‖] David F. Plusquellic,[⊥] Timothy S. Zwier,[§,#] and Stephen Drucker[*,†]

†Department of Chemistry and Biochemistry, University of Wisconsin-Eau Claire, 105 Garfield Avenue, Eau Claire, WI 54701

‡Current address: Department of Chemistry, University of Southern California

¶Current address: College of Pharmacy, University of Michigan

§Department of Chemistry, Purdue University, 560 Oval Drive, West Lafayette, IN 47907

‖Current address: Department of Chemistry, Massachusetts Institute of Technology

⊥Applied Physics Division, National Institute of Standards and Technology, 325 Broadway Avenue, Boulder, CO 80305

#Current address: Combustion Research Facility, Sandia National Laboratories

E-mail: druckers@uwec.edu

Phone: (715) 836-5390

Figs. 1S and 2S show origin-band contours for the $T_1(n,\pi^*) \leftarrow S_0$ transition of 4PN, measured using 3-atm and 1-atm expansions of helium, respectively. Also shown are simulations using the two-temperature model descibed in the main text. Both simulations use the molecular parameters listed in Table 4 of the main text. These figures complement the band contour shown in the main text (Fig. 8) for a 2-atm helium expansion. The agreement between simulated and observed contours, shown here and in the main text, indicates that the two-temperature model is adequate for characterizing the jet expansion over a modest range of backing pressures.

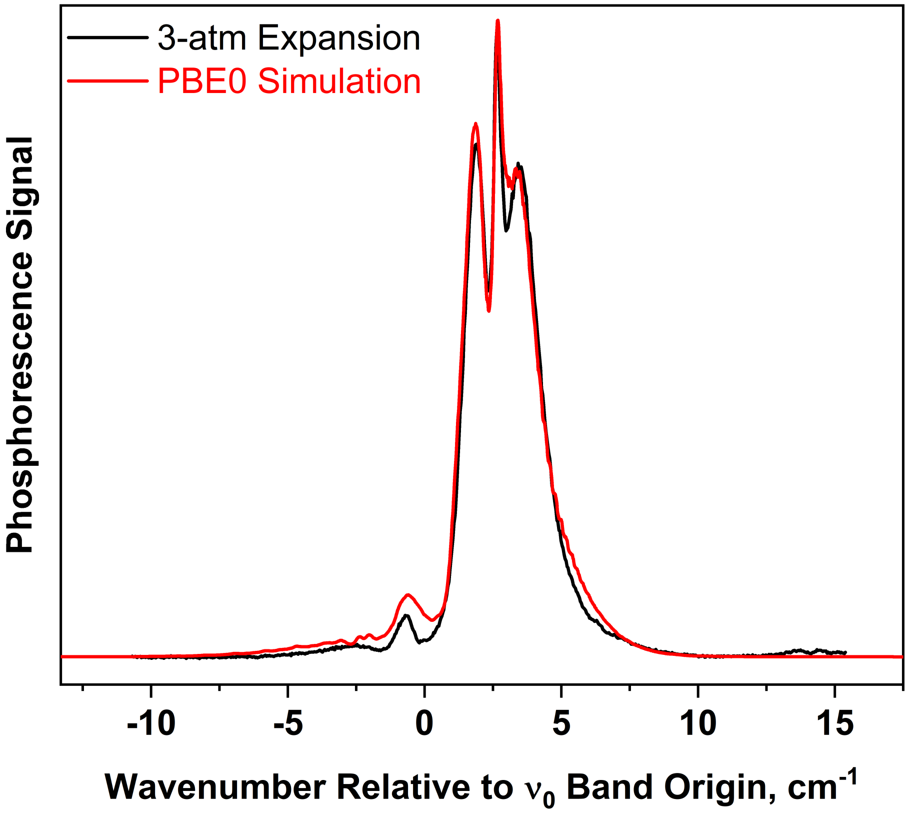

Figure 1S: Jet-cooled $T_1(n,\pi^*) \leftarrow S_0$ phosphorescence excitation spectrum of 4PN, recorded using a helium backing pressure of 3 atm (black trace). The simulated spectrum (red trace) was generated using the same molecular constants as in Fig. 8 of the main text, but with rotational temperatures of 3 K and 13 K and weighting factors of 0.6 and 0.4, respectively.

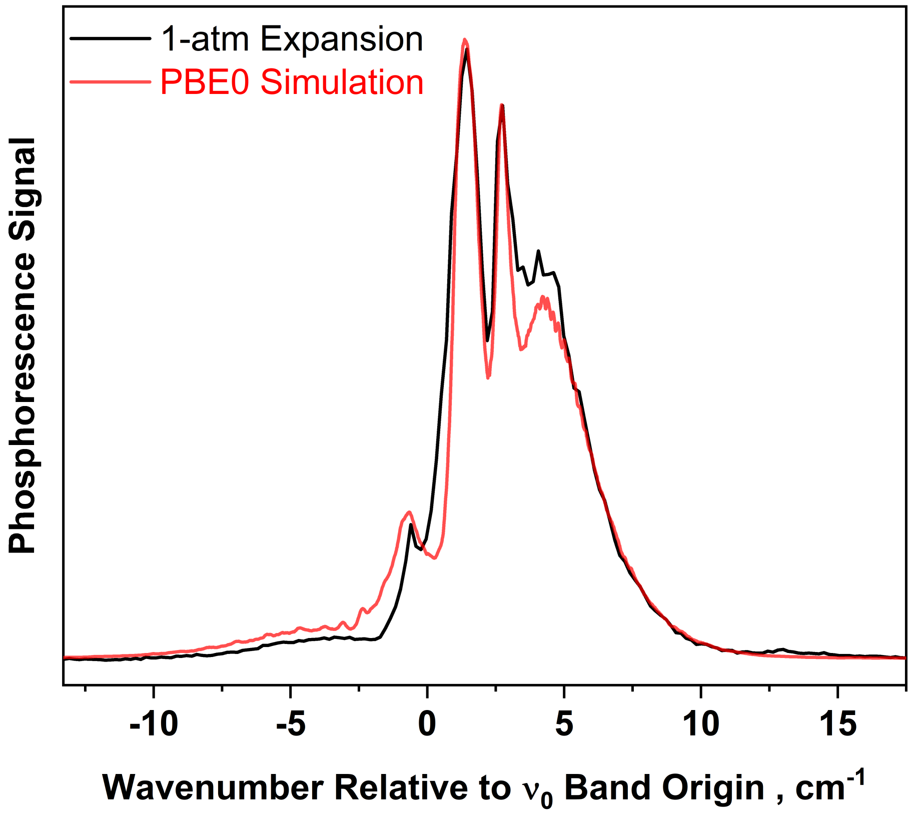

Figure 2S: Jet-cooled $T_1(n,\pi^*) \leftarrow S_0$ phosphorescence excitation spectrum of 4PN, recorded using a helium backing pressure of 1 atm (black trace). The simulated spectrum (red trace) was generated using the same molecular constants as in Fig. 8 of the main text, but with rotational temperatures of 13 K and 23 K and weighting factors of 0.4 and 0.6, respectively.
